# Network community, clusters and hubs in cortical micro circuits

**DOI:** 10.1186/1471-2202-15-S1-F2

**Published:** 2014-07-21

**Authors:** Masanori Shimono, John M Beggs

**Affiliations:** 1 Department of Physics, University of Indiana, Bloomington, IN, 47405, USA

## 

Networks of cortical neurons are essentially non-random [1]. Although it is known that such networks show interesting structure at multiple temporal and spatial scales [2], almost no experimental work has been done to reveal how structures at these different scales relate to each other.

**Figure 1:**
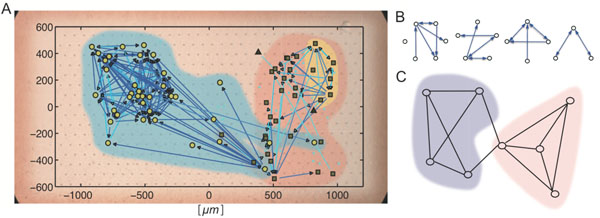
(A) An example of spatial distribution of neurons and effective connections. Different markers indicate different communities. The biggest two communities are covered by blue and red regions. Upper-right yellow region is an example cluster of 6 neurons. (B) Examples of clusters of 3-6 neurons. (C) An illustration of community structures. Connections are relatively denser among neurons within each community and sparser between neurons in different communities.

This study aimed to clarify important relations between non-randomness in groups of 3-6 neurons (clusters) and non-randomness in groups of 50-100 neurons (communities) through five steps. First, we recorded spontaneous activity of up to 500 neurons from rodent somatosensory cortex using a 512ch. multi-electrode system over one hour [3]. Second, we reconstructed effective connectivity using transfer entropy [4]. Third, we compared topologies of effective networks at the 3-6 neuron scale (clusters including motifs [Figure1-B]) with topologies of synaptic connections measured from 12 neuron simultaneous patch clamp experiments [5,6]. Fourth, we constructed community or modular structures representing non-randomness from larger groups of neurons. Fifth, we evaluated the extent to which structure at each of these scales was robust. We did this by swapping connections from high degree nodes (hubs) with those from low degree nodes (non-hubs).

We found three things. First, the degree-distribution followed a power-law This demonstrated that hubs could not have been the result of random sampling from a Gaussian distribution. Second, effective networks consisting of hundreds of cortical neurons have distinctive non-random structures of connectivity at two different scales. Third, structure at the cluster level was relatively more fragile than structure at the community level. The difference between non-randomness evaluated by cluster and community will become the important first step to understand multiple different scales of cortical neuronal networks.
